# Genome-Wide Association Study of Metabolic Traits Reveals Novel Gene-Metabolite-Disease Links

**DOI:** 10.1371/journal.pgen.1004132

**Published:** 2014-02-20

**Authors:** Rico Rueedi, Mirko Ledda, Andrew W. Nicholls, Reza M. Salek, Pedro Marques-Vidal, Edgard Morya, Koichi Sameshima, Ivan Montoliu, Laeticia Da Silva, Sebastiano Collino, François-Pierre Martin, Serge Rezzi, Christoph Steinbeck, Dawn M. Waterworth, Gérard Waeber, Peter Vollenweider, Jacques S. Beckmann, Johannes Le Coutre, Vincent Mooser, Sven Bergmann, Ulrich K. Genick, Zoltán Kutalik

**Affiliations:** 1 Department of Medical Genetics, University of Lausanne, Lausanne, Switzerland; 2 Swiss Institute of Bioinformatics, Lausanne, Switzerland; 3 Department of Food-Consumer Interaction, Nestlé Research Center, Lausanne, Switzerland; 4 Investigative Preclinical Toxicology, GlaxoSmithKline R&D, Ware, Herts, United Kingdom; 5 European Bioinformatics Institute, Wellcome Trust Genome Campus, Hinxton, Cambridge, United Kingdom; 6 Department of Biochemistry & Cambridge Systems Biology Centre, University of Cambridge, Cambridge, United Kingdom; 7 Institute of Social and Preventive Medicine (IUMSP), Centre Hospitalier Universitaire Vaudois (CHUV), University of Lausanne, Lausanne, Switzerland; 8 Sensonomic Laboratory of Alberto Santos Dumont Research Support Association and IEP Sirio, Libanes Hospital, São Paulo, Brazil; 9 Edmond and Lily Safra International Institute of Neuroscience of Natal, Natal, Brazil; 10 Department of Radiology and Oncology, Faculdade de Medicina, Universidade de São Paulo, São Paulo, Brazil; 11 Department of Bioanalytical Sciences, Nestlé Research Center, Lausanne, Switzerland; 12 Medical Genetics, GlaxoSmithKline, Philadelphia, Pennsylvania, United States of America; 13 Department of Medicine, Internal Medicine, Centre Hospitalier Universitaire Vaudois (CHUV), Lausanne, Switzerland; 14 Service of Medical Genetics, Centre Hospitalier Universitaire Vaudois (CHUV), Lausanne, Switzerland; 15 Organization for Interdisciplinary Research Projects, The University of Tokyo, Yayoi, Bunkyo-ku, Tokyo, Japan; 16 Department of Medicine, Centre Hospitalier Universitaire Vaudois (CHUV), Lausanne, Switzerland; Georgia Institute of Technology, United States of America

## Abstract

Metabolic traits are molecular phenotypes that can drive clinical phenotypes and may predict disease progression. Here, we report results from a metabolome- and genome-wide association study on ^1^H-NMR urine metabolic profiles. The study was conducted within an untargeted approach, employing a novel method for compound identification. From our discovery cohort of 835 Caucasian individuals who participated in the *CoLaus* study, we identified 139 suggestively significant (*P*<5×10^−8^) and independent associations between single nucleotide polymorphisms (SNP) and metabolome features. Fifty-six of these associations replicated in the *TasteSensomics* cohort, comprising 601 individuals from São Paulo of vastly diverse ethnic background. They correspond to eleven gene-metabolite associations, six of which had been previously identified in the urine metabolome and three in the serum metabolome. Our key novel findings are the associations of two SNPs with NMR spectral signatures pointing to fucose (rs492602, *P* = 6.9×10^−44^) and lysine (rs8101881, *P* = 1.2×10^−33^), respectively. Fine-mapping of the first locus pinpointed the *FUT2* gene, which encodes a fucosyltransferase enzyme and has previously been associated with Crohn's disease. This implicates fucose as a potential prognostic disease marker, for which there is already published evidence from a mouse model. The second SNP lies within the *SLC7A9* gene, rare mutations of which have been linked to severe kidney damage. The replication of previous associations and our new discoveries demonstrate the potential of untargeted metabolomics GWAS to robustly identify molecular disease markers.

## Author Summary

The concentrations of small molecules known as metabolites, are subject to tight regulation in all organisms. Collectively, the metabolite concentrations make up the metabolome, which differs amongst individuals as a function of their environment and genetic makeup. In our study, we have further developed an untargeted approach to identify genetic factors affecting human metabolism. In this approach, we first identify all genetic variants that correlate with any of the measured metabolome features in a large set of individuals. For these variants, we then compute a profile of significance for association with all features, generating a signature that facilitates the expert or computational identification of the metabolite whose concentration is most likely affected by the genetic variant at hand. Our study replicated many of the previously reported genetically driven variations in human metabolism and revealed two new striking examples of genetic variations with a sizeable effect on the urine metabolome. Interestingly, in these two gene-metabolite pairs both the gene and the affected metabolite are related to human diseases – Crohn's disease in the first case, and kidney disease in the second. This highlights the connection between genetic predispositions, affected metabolites, and human health.

## Introduction

Genome-wide association studies (GWAS) search for associations between phenotypes and common variants within large collections of samples [1]. These studies usually focus on organismal phenotypes [2]–[6]. Recently however, molecular phenotypes, including gene-expression [7], [8] and metabotypes [9]–[14], have also been investigated. Studying the effects of genetic variations on molecular phenotypes is motivated by two characteristics common to the vast majority of GWAS on organismal phenotypes: first, the biological mechanisms underlying the associations are often unknown; and second, the significantly associated loci individually explain only a small fraction of variability of the organismal phenotype, and even cumulatively fall far from explaining the estimated heritability of the phenotype [15]. Molecular phenotypes can be considered as far less removed from the primary causal variants. In agreement with this, GWAS on these phenotypes uncover associations generally characterized by larger effect sizes and higher explained variances. For example, the study of gene expression data from different tissues revealed hundreds of SNPs explaining a significant portion (>5%) of the gene expression levels of (usually) neighboring genes. These *expression quantitative trait loci* (eQTL) overlaid with GWAS hits for organismal phenotypes reveal significant enrichment [16], hinting at the underlying causal biological mechanisms. Large effect sizes have also been observed for many *metabolic quantitative trait loci* (mQTL) (see [17] for a recent review). Indeed, several metabolite concentrations measured in urine or serum are genetically determined in a close-to-monogenic manner [10], [12], [18]. More recently, mQTLs have been studied in more depth in the context of organismal phenotypes in order to develop potential prognostic disease markers [11], [19].

The technologies used to measure the metabolome (generally mass spectrometry or NMR spectroscopy) produce high-dimensional raw data. Most GWAS for mQTLs employ estimates of metabolite concentrations that have been derived from these data after normalization. This data transformation is far from trivial, and is performed only for a subset of at most a few hundred metabolites of the much larger set of known human metabolites. The non-transformed data are ignored in the subsequent GWAS, so that this *targeted* approach to mQTL GWAS discards potentially valuable raw data captured by the analytical technique. In our study, we followed an *untargeted* approach, similar to the one previously used in the analysis of rodent [20], [21] and human metabolism [22]. In this approach, instead of seeking to transform normalized data into metabolite concentrations as target traits for GWAS, we use the normalized data themselves as phenotypes to be associated with the genotypes, thereby pinpointing metabolome features from these data that have a genetic association. The subsequent identification of metabolites is attempted only using these features, and thereby focused on compounds whose concentrations have a significant genetic determinant.

## Results

**Figure 1:**
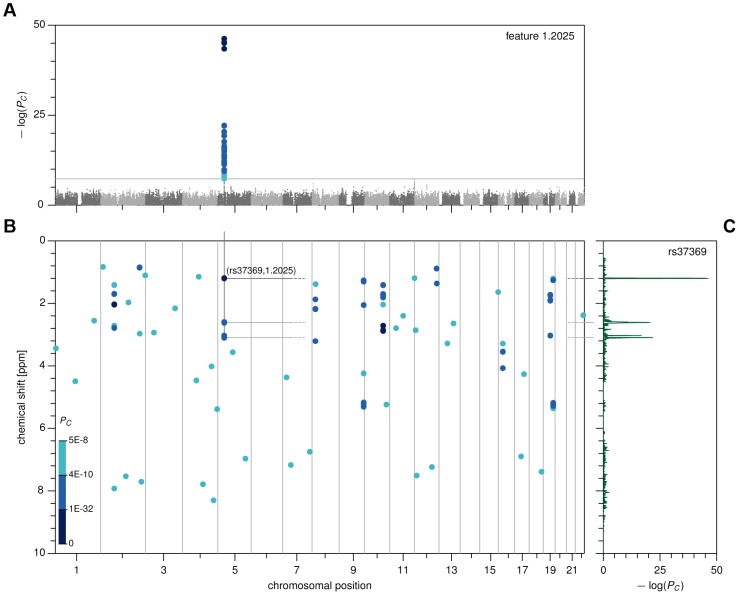
Genome- and metabolome-wide analysis results, first stage. (A) Manhattan plot for feature 1.2025. (B) Genome- and metabolome-wide P-value heat map, showing associations with *P_C_*<5×10^−8^ in *CoLaus*. (C) Pseudo-spectrum for SNP rs37369, obtained by plotting the association P-values between rs37369 and all metabolic features.

Our study concerns metabolites in urine samples, measured by ^1^H-NMR spectroscopy (details on sample preparation and spectrum acquisition are provided in the Materials and Methods section). We binned the ^1^H-NMR spectra into approximately 2,000 uniform bins, and defined the average intensity of the NMR signal in a bin as a *metabolome feature*. In our untargeted approach, we used these features—which, combined, contain the full spectroscopic data—as molecular phenotypes. After quality filtering (Materials and Methods), we maintained 1,276 of these features for subsequent analysis. We then followed a two-stage GWAS design, wherein we tested all possible SNP-feature pairs for association in the *Cohorte Lausannoise*, or *CoLaus* (see figure 1A for the Manhattan plot corresponding to a single feature, figure S1 for a three-dimensional illustration of Manhattan plots for all features, and figure 1B for the P-value heat map summarizing only the significant associations). After pruning according to SNP linkage and feature correlation, pairs indicating suggestively significant association (P-value below 5×10^−8^) in *CoLaus* (N = 835) were tested for replication in the *TasteSensomics* cohort [23], [24] (N = 601). Out of 139 discovered independent associations, 56 replicated (see table S1 for detailed list).

For this manageable set of reproducible associations, we then sought to identify the underlying metabolites. To this end, we devised a method that we call *metabomatching*. Our method makes use of the fact that the NMR spectrum of most metabolites comprises multiple peaks, so that the genetic effect of a SNP on a metabolite usually results in associations of that SNP with multiple metabolome features. This concept is best visualized by way of the *pseudo-spectrum* of a SNP (see figure 1C for an example), consisting of the set of significance values (−log(P-values)) of its associations with each of the 1,276 features. We observed that in cases where the genetic effect is sufficiently strong, the pseudo-spectrum tends to be similar to the NMR spectrum of the underlying metabolite, allowing its identification.

**Figure 2:**
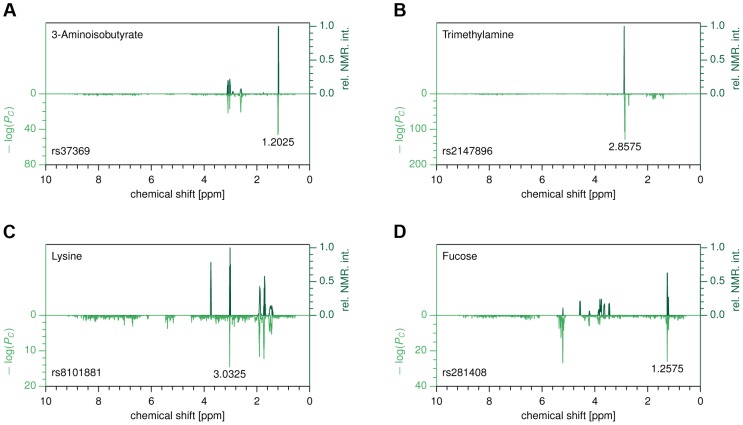
Metabomatching. Each subfigure compares the *CoLaus* pseudo-spectrum (bottom half) with the NMR spectrum (top half) of the most likely candidate for the associated metabolite. (A) rs37369 vs. 3-aminoisobutyrate. (B) rs2147896 in *PYROXD2* vs. trimethylamine (C) rs8101881 in *SLC7A9* vs. lysine (D) rs281408 in *FUT2* vs. fucose.

Specifically, for a given SNP, metabomatching assigns scores to all metabolites with known NMR spectrum. The scores are computed using the significance values of the features that correspond to peaks in the known spectra (see Materials and Methods for details). The metabolites are then ranked, based on these scores, to identify the candidate metabolites most likely to underlie the association. As an example, for SNP rs37369, the top-ranked candidate metabolite is 3-aminoisobutyrate, thereby replicating the association found in previous metabolomics studies [11], [12], [22]. Figure 2A shows how closely the NMR spectrum of 3-aminoisobutyrate (upper half) matches the pseudo-spectrum of rs37369 (lower half).

In order to evaluate the robustness of the metabomatching method, we collected all known metabolites whose concentrations in urine had previously been found to be associated with SNPs by the two largest-to-date studies [12], [22]. Among these established SNP-metabolite pairs, we then considered only those for which our association P-values are below 10^−6^ and whose metabolites have a known NMR spectrum (see table S2). For these controls, metabomatching proved very efficient in selecting the reference compounds, which ranked within the top 1% for 5 out of 7 testable associations, and within the top 10% for the remaining two (see figure 2A–C and figure S2). Encouraged by these findings, we decided to use metabomatching to identify the metabolites (or metabolite families) underlying some of our associations.

Grouping features by metabolites and SNPs by genetic loci, we reduced our 56 SNP-feature associations to 11 locus-metabolite associations, listed in table 1. We replicated the previously published urine associations of *ALMS1* with N-acetylated compounds (figure S2A), *AGXT2* with 3-aminoisobutyrate (figure 2A), and *PSMD9* with 2-hydroxyisobutyrate (figure S2D). For *PYROXD2*, we replicated the association with trimethylamine (figure 2B), but also found associations with several features not part of the spectrum of trimethylamine, suggesting that one or more additional metabolites could be implicated. Similarly, the published association of *NAT2* is with the formate-succinate ratio [12], but neither of these compounds contains the features implicated by our association (Figure S2C). For the associations of SNPs in *ACADL*, *ABO*, and *ACADS*, linked SNPs have been found to associate with metabolite concentrations in serum. However, without conclusive identification of the metabolites underlying the associated features we could not determine whether our associations are the exact urine analogs of known serum associations, or whether they involve novel or related metabolites.

**Table 1:** Locus-metabolite associations.

Locus				Metabolite		Association				Published (Body fluid)	Organismal Phenotype
Gene	SNP	Chr	Position	Compound	Feature(s)	*x_C_*	*x_T_*	*x_m_*	*P_m_*		
*ALMS1*	rs11884776	2	73,600,431	N-acetylated compounds	1.6975/**2.0375**/2.7875	1.08	0.96	1.02	3.4×10^−209^	Urine [22]	Kidney disease
*ACADL*	rs3764913	2	210,783,154	Unknown	**0.8475**	0.41	0.27	0.36	2.9×10^−19^	Serum [10]	
*AGXT2*	rs37370	5	35,075,243	3-Aminoisobutyrate	1.1975/**1.2025**/2.6075/2.6125/2.6275/3.0275/3.0925–3.1075	1.05	0.81	0.94	1.2×10^−65^	Urine [12]	
*NAT2*	rs4921914	8	18,316,718	Unknown	**2.1875**	0.60	0.44	0.51	4.4×10^−32^	Urine (Ratio) [12]	Bladder cancer
*ABO*	rs579459	9	135,143,989	Unknown	1.2975/2.0525/4.2375/5.1625/**5.1825/**5.2625	0.52	0.55	0.53	1.8×10^−32^	Serum [11]	Pancreatic cancer, CHD, Venous thromboembolism
*PYROXD2*	rs2147896	10	100,138,166	Trimethylamine	**2.8575**–2.8825	−0.96	−0.68	−0.85	2.6×10^−164^	Urine [22]	
*PYROXD2*	rs4345897	10	100,137,050	Unknown	1.7775/**1.8025**/2.7125	−0.41	−0.29	−0.36	4.5×10^−21^	New	
*ACADS*	rs3916	12	119,661,655	Unknown	**0.8875**	0.46	0.33	0.40	2.4×10^−22^	Serum [10], [11]	
*PSMD9*	rs7314056	12	120,827,347	2-Hydroxyisobutyrate	**1.3625**	−0.46	−0.41	−0.44	4.0×10^−16^	Urine [12]	
*SLC7A9*	rs8101881	19	38,056,468	Lysine	1.7325/1.9025/**3.0325**	0.39	0.54	0.45	1.2×10^−33^	Urine (Ratio) [12]	Kidney disease
*FUT2*	rs492602	19	53,898,229	Fucose	**1.2575**/5.2125/5.2275/5.2825	0.71	0.54	0.60	6.9×10^−44^	New	Crohn's disease

For every locus, the association results are listed for the strongest association, after meta-analysis, of a SNP in the locus with a feature (bold) of the metabolite. Abbreviations: Chr – chromosome, Position – chromosomal position in NCBI build 36, *x_C_* – effect size in *CoLaus*, *x_T_* – effect size in *TasteSensomics*, *x_m_* – effect size after meta-analysis, *P_m_* – P-value after meta-analysis.

In the traditionally applied SNP-pruning procedure, focus is given only to the most significant SNP and the phenomenon of (semi-)independent contribution of adjacent SNPs (termed as *allelic heterogeneity*) is ignored. To overcome this limitation, we tested for allelic heterogeneity for each of our 11 locus-feature pairs using multivariate association [25], [26]. We found evidence for secondary signals for four of these pairs in the *CoLaus* sample, and for two of them, both involving the *AGXT2* locus, allelic heterogeneity was replicated in the *TasteSensomics* cohort (table 2). For these replicating cases, the variance explained by the multiple SNP association was up to 50% greater than that of the single SNP association, demonstrating the importance of allelic heterogeneity, still often overlooked in GWAS [26].

**Table 2:** Allelic heterogeneity at the *AGXT2* locus.

Locus		*CoLaus*							*TasteSensomics*						
Chr	Position	Feature	SNP	*P_C_*	*x_C_*	*R^2^*	*R^2^_diff_*	model P	Feature	SNP	*P_T_*	*x_T_*	*R^2^*	*R^2^_diff_*	model *P*
5	34,537,671–35,578,717	1.2025	rs37370	2.1×10^−37^	0.95	0.278	0.079	2.0×10^−4^	1.204	rs37370	2.2×10^−21^	0.92	0.130	0.047	2.0×10^−4^
			rs7717823	1.1×10^−20^	−0.47					rs455423	5.9×10^−8^	−0.41			
			rs6880595	5.1×10^−4^	0.18										
5	34,537,671–35,578,717	3.0975	rs37369	3.6×10^−16^	0.78	0.115	0.023	2.2×10^−3^	3.096	rs37370	6.6×10^−12^	0.68	0.097	0.026	6.2×10^−3^
			rs7717823	3.5×10^−6^	−0.25					rs455423	1.0×10^−4^	−0.31			

Abbreviations: *P_C_, P_T_* – P-values, *x_C_, x_T_* – multivariate effect sizes, *R^2^* – explained variance of full model, *R^2^_diff_* – additional explained variance of full model compared to best single SNP association, *model P* – probability of observing same or equal *R^2^_diff_* increase with the same stepwise model selection for 2,500 permuted phenotypes.

**Figure 3:**
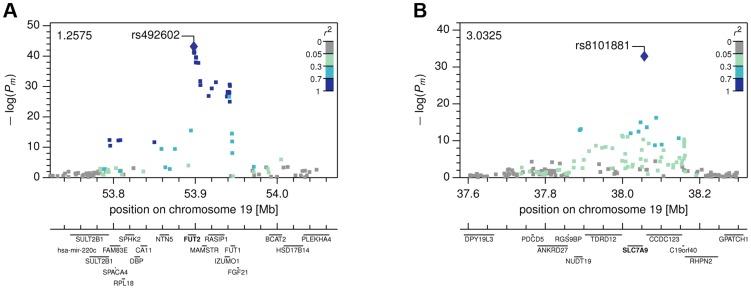
Local Manhattan plots. The Manhattan plots show combined −log(P-values) in the neighborhood of the most strongly associated SNP for (A) the *FUT2* with fucose association, and (B) the *SLC7A9* with lysine association.

**Figure 4:**
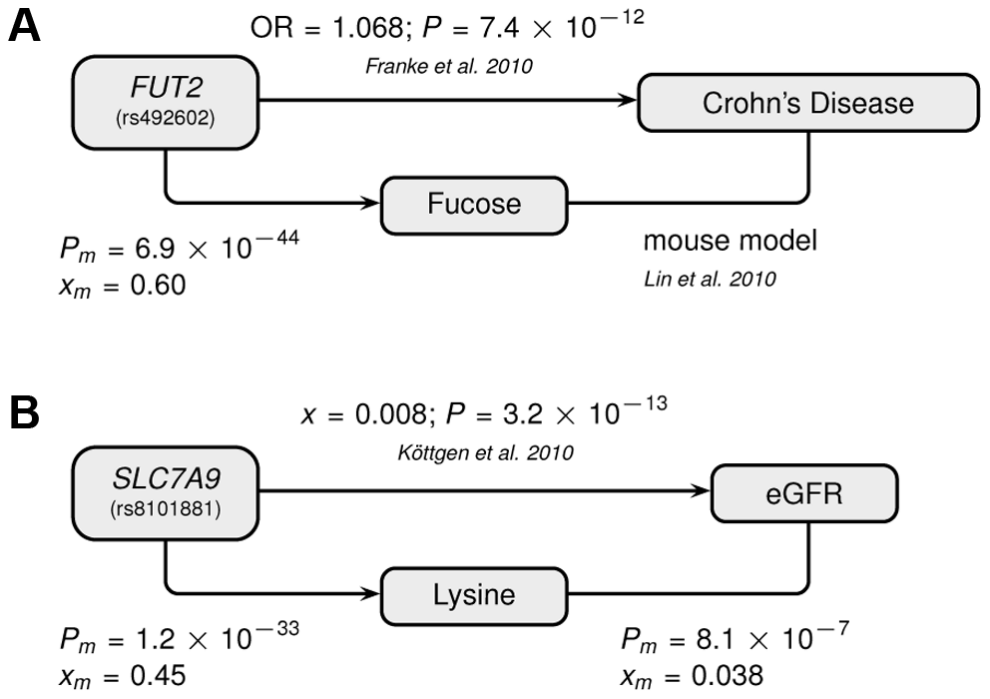
Genotype-Metabotype-Phenotype associations. The two novel gene-metabolite associations of this study implicate SNPs that had previously been associated with disease-related phenotypes by the indicated publications: (A) Fucose–Crohn's disease–*FUT2* (rs492602), (B) Lysine–eGFR–*SLC7A9* (rs8101881). A link between the metabolite and the phenotype has been established for (A) based on a mouse model and for (B) by a direct correlation with the indicated significance and effect size. Abbreviations: OR refers to the odds ratio, *x* to the linear regression effect size, *P* to the corresponding P-value, and the *m*-index indicates values obtained in the combined *CoLaus* and *TasteSensomics* sample.

For our first novel association, metabomatching allowed the identification of the underlying metabolite. As illustrated in figure 2D, the pseudo-spectrum of rs281408 (lower half) closely resembles the NMR spectrum (upper half) of the top-ranked candidate, fucose. We confirmed this *in-silico* identification using NMR spectroscopy of fucose-spiked urine samples. In *CoLaus*, the SNPs associated with fucose fall within a large LD block on chromosome 19 encompassing the *FUT2*, *RASIP1*, and *IZUMO1* genes. However, the *TasteSensomics* population has a different genetic structure within this region (figure S3), such that the combined association signal, led by rs492602 (*r^2^* = 0.87 with rs281408), could be narrowed down to *FUT2* specifically (Figure 3A). *FUT2* encodes a fucosyltransferase enzyme that is essential for the secretion and display of ABO blood group antigens on mucosal surface cells. Mucosal ABO-antigens serve as attachment points for both beneficial gut bacteria and harmful viruses [27], [28], which is thought to have driven the complex evolution of *FUT2* [29]. In addition, fucose, the substrate of the fucosyltransferase enzyme, was shown to impact human gut microbial composition [30], [31], and thereby gut health [32], [33]. The role of *FUT2* in gut microbial ecology is further substantiated by the association of its SNP rs281379 (*r^2^* = 0.76 with rs492602) with Crohn's disease (CD), as found in a sample of over 50 K individuals [34] (figure 4A). Several urinary metabolites (not including fucose) were shown to distinguish between inflammatory bowel disease patients (including those with CD) and healthy subjects [35]. Moreover, significantly elevated fucose levels in urine were found in mice with an interleukin-10 deficiency, the mouse model of CD [36], [37]. This *FUT2*-independent link between urinary fucose levels and CD may be indicating that the elevated urine fucose levels, also observed in human *FUT2* non-secretors, do not simply result from the elimination of fucose that was not secreted into the mucosal layers. Instead this elevation may be a consequence of (and metabolic indicator for) early sub-symptomatic changes from a healthy gut flora towards the dysbiosis of CD. While its exact role is unclear, fucose is certainly an interesting candidate for further exploration of the metabolic causes and effects of CD, or inflammatory bowel disorders in general.

Our second novel association links the SNP rs8101881 with a metabolite identified as lysine by our metabomatching method (figure 2C). This SNP falls within the *SLC7A9* gene (in a different region of chromosome 19, see Figure 3B). SNPs at this locus have already been found to be significantly associated with the lysine/valine ratio [12], but not lysine alone. *SLC7A9* is linked to kidney function: rare mutations in *SLC7A9* cause severe kidney damage [38], and a common variant (rs12460876, linked to rs8101881 with *r^2^* = 0.996) is associated with the estimated glomerular filtration rate (eGFR) [39], which is a key clinical measure of kidney health. Interestingly, lysine concentration shows a strong association with eGFR in the combined *CoLaus* and *TasteSensomics* sample (*x_m_* = 0.038, SE = 0.008, *P_m_* = 8.1×10^−7^), regardless of the rs8101881 genotype. To further explore these links (figure 4B) we used Mendelian randomization (MR) [40], [41] in order to assess whether lysine levels may be causative for chronic kidney disease. We employed rs8101881 as instrument (F-statistic = 46.22) and the tests proposed by Glymour *et al.* [42] indicated no violation of the assumptions of MR. We then computed the two-stage least-squares (2SLS) estimate as done by Ehret et al. [2], where the rs8101881-lysine effect was calculated combining the results from the *CoLaus* and *TasteSensomics* cohorts, while the effect of rs8101881 on eGFR was estimated using CKDGen [39] summary statistics. Although the 2SLS estimate was consistent (overlapping in confidence interval) with the ordinary least-squares (OLS) estimate of lysine on eGFR (*x_m_* = 0.038), it was non-significant (*x* = 0.02, *P* = 0.54), hence we have no sufficient evidence to claim a causal effect of lysine levels on eGFR.

## Discussion

We conducted a genome- and metabolome-wide association study of untargeted NMR data to reveal novel SNP-feature associations. Using both manual and automated annotation, we identified the metabolites underlying more than half of the discovered associations.

The high number of associations found to replicate (56 out of 139) is indicative of the robustness of mQTL GWAS in general, and our feature-based approach in particular. Our discovery and replication cohorts have different population origins —European for the Swiss cohort *CoLaus*, genetically admixed, from African, European, and Asian founders, for the Brazilian cohort *TasteSensomics*—indicating that the genetic effects on the metabotypes are likely to be both ethnicity-independent, and robust against potential variations of diet and other environmental factors.

The two metabolomic data sets we used for discovery and replication were collected independently, initially without the intention of combining them. As a result, the respective experimental conditions were not always well matched (see Materials and Methods for details). Since differences in the experimental setups can cause significant changes in the chemical shifts of specific metabolite absorption bands, one could have expected that this would cause a significant problem to our feature-based approach. Yet in practice, this did not appear to be a significant impediment, given the high rate of replication between our two studies. This indicates that the feature-based approach is rather robust against variations in experimental conditions. The reliability of the feature-based approach is further evidenced by the high overlap between our associations and previously described results [11], [12], [22].

In comparison to previous targeted approaches, where metabolite identification is applied before GWAS, the feature-based approach has two main advantages. The first, and most important one, is that by moving the identification of metabolite concentrations after the association phase, the complete metabolomic data captured by spectroscopy are analyzed. As a consequence, the feature-based approach can potentially provide additional association signals that would have been missed by a targeted approach.

The second advantage, which is of a more pragmatic nature, is that the burden associated with metabolic identification is considerably reduced. Indeed only the metabolites of interest, namely those found to have a genetic component, need identification. Even so, identification of all metabolites of interest can prove difficult, and cases may exist where identification will require further experimental work (like the collection of two-dimensional homo- and heteronuclear NMR spectra, for example). Such additional analysis was precluded in our study due to the destruction of samples after ^1^H-NMR analysis in accordance with study protocols and informed consent.

A key message of our study is that our metabomatching method may be useful for other cohort-based metabolomics projects when resources for compound identification in terms of material or expert time are limited. Essentially, the information inherent in the GWAS signals can complement (and sometimes even replace) traditional sample-based metabolite identification. As the information in databases of NMR spectra of individual metabolites increases, the method may become a powerful strategy for metabolite identification in GWAS involving untargeted metabolomics.

In summary, the replication of locus-metabolite associations with previous studies [9]–[13] and the unequivocal identification of two new gene-metabolite associations indicate that the feature-based approach, combined with pseudo-spectrum based identification, is a reliable approach for metabolome- and genome-wide association studies. In cases where newly identified association signals are of marginal strength, metabolite identification may be followed-up by model-based quantification of the metabolite [43], [44] to potentially improve the association signal, and provide a more accurate effect size estimate. While the assignment to metabolites of all associated features can require substantial follow-up work, this may not be necessary if the primary objective of a study was to elucidate novel genetic loci relevant for general metabolomic variability. Specifically, while associations with unidentified metabolites may lack a direct mechanistic interpretation, they can still prove to be valuable biomarkers of certain clinical phenotypes [45], [46]. Finally, the unidentified metabolite underlying an association may correspond to an *unknown metabolite* in the sense, used in Krumsiek *et al.* [47], of “a molecule which can reproducibly be detected and quantified […] but whose chemical identity has not been elucidated”, in which case the genetic association itself may provide identifying information.

Our GWAS revealed two new SNP-metabolite associations of potential clinical relevance. We found urine fucose concentration to be associated with variants in the *FUT2* gene, which is linked to gut microbial ecology in general, and to Crohn's disease in particular. Furthermore, we found urine lysine concentration to be associated with SNPs in the *SLC7A9* gene, which is linked to kidney function and to kidney failure specifically. We confirmed the link to kidney function with a significant lysine-eGFR association. Our Mendelian randomization was inconclusive for a causal link between urine lysine levels and eGFR (as a measure of kidney filtering capacity). Yet, we only had about 12% power and a sample size of at least 11,400 would be required for providing a conclusive answer (i.e. having over 80% power). Molecular trait association can not only help us to better understand the underlying biological processes, but also shed light on the interplay between genetic predisposition and environmental factors. In our case, figuring out how lysine levels are influenced by diet may thus help to develop nutritional intervention programs to counter kidney problems before they manifest themselves in a clinical phenotype. In summary, this study provided specific evidence that genetically influenced metabolite concentrations can play a crucial role in disease progression, and that these metabolites may provide an avenue for better diagnosis and prevention of diseases.

## Materials and Methods

For the *Cohorte Lausannoise (CoLaus)* study, genotyping was performed using the Affymetrix GeneChip Human Mapping 500 K array set. Genotypes were called using BRLMM [48]. Duplicate individuals, and first and second degree relatives, were identified by computing genomic identity-by-descent coefficients, using PLINK [49]. The younger individual from each duplicate or relative pair was removed. Individuals with call rate below 90% were excluded from further analysis. The full set of unmeasured HapMap II SNPs (release 21) was imputed using 390,631 measured SNPs (with Hardy-Weinberg P-value above 10^−7^ and MAF above 1%). Imputation was performed using IMPUTE [50] version 0.2.0. Expected allele dosages were computed for 2,557,249 SNPs.

For the *TasteSensomics* study, genotyping was performed on the Illumina Human Omni-Quad1 platform. Genotype calling was performed with Beadstudio software (Illumina). Calls with a genotyping score below 0.2 were excluded from further analysis. SNPs with a call rate below 90% and individuals with a call rate below 95% were also excluded, leaving 989,972 available SNPs, with an overlap of 713,870 SNPs with the *CoLaus* cohort. No imputation was performed in this cohort, since none of the available HapMap panels were considered as sufficiently representative for the admixed population investigated in this study.

In the *CoLaus* cohort, 974 individuals each provided 1 urine sample for metabolic analysis. The *CoLaus* study was approved by the Institutional Ethics Committee of the University of Lausanne. All study participants gave written consent including for genetic studies. Prior to urinalysis, samples were stored at −80°C. Each sample was comprised of 400 µL urine and 200 µL of a 0.2M deuterated phosphate buffer solution (pH 7.4). Samples were centrifuged to remove precipitates, and to 500 µL aliquots of the resulting supernatant, 100 µL of a solution of 0.1% (w/v) sodium trimethylsilyl propionate (TSP) and 1% (w/v) sodium azide in D_2_O was added. The TSP provided a chemical shift reference (δ0.0), the sodium azide acted as a bactericide, and the D_2_O provided a deuterium field-frequency lock signal for the NMR spectrometer. ^1^H NMR spectra were acquired at 300 K on a Bruker Avance II 700 MHz spectrometer (Bruker Biospin, Rheinstetten, Germany) using a standard ^1^H detection pulse sequence with water suppression.

In the *TasteSensomics* cohort, 601 individuals donated 3 samples each over a period of 2 weeks. 3 mM sodium azide was added to the samples to prevent microbial growth. Samples were then frozen and stored at −80°C prior to urinalysis. Urine aliquots of 400 µL were adjusted to pH 6.8 using 200 µL of deuterated phosphate buffer solution (final concentration of 0.2M) containing 1 mM of sodium TSP. ^1^H NMR spectra were recorded at 300 K on a Bruker Avance II 600 MHz spectrometer, using a standard ^1^H detection pulse sequence with water suppression.

*CoLaus* ^1^H spectra were binned in chemical shift increments of 0.005 ppm, resulting in metabolic profiles of 2,200 metabolome features. Filtering out features then samples with more than 5% of missing values, a dataset composed of 1,276 features for 835 individuals was obtained. *TasteSensomics* ^1^H spectra were binned in increments of 0.0032 ppm, resulting in profiles of 2,400 features. More sophisticated binning procedures, such as adaptive binning [51], [52], could have been applied, but standard uniform binning has been shown to be successful by us [53], [54] and others [55], [56]. Bin intensities were log-averaged across replicate samples for each individual, and spectral qualities were such that all features and subjects were included in the analysis. For each individual, we applied a Z-score transformation in order to achieve zero mean and unit variance. This statistical normalization yields metabolic profiles similar to those resulting from common biological normalizations, such as normalization by total metabolite content (median correlation *r* = 0.92), or normalization by urinary creatinine measured before freezing and thawing (resulting in lower median correlation *r* = 0.45).

In addition to the standard confounding factors that are age, sex, post-menopausal status, and the principal components of the genotype, metabolic profiles are sensitive to lifestyle factors, dietary behavior, and creatinine levels. Among the 36 such factors available for the *CoLaus* sample, we select those which associated with at least 2% of the features, resulting in the 12 factor subset comprising age, sex, post-menopausal status, the 1^st^ principal component of the genotype, the 2^nd^ and 4^th^ principal components of the dietary profile, smoking behavior, caffeine intake, alcohol intake, physical activity, urinary creatinine, and serum creatinine. For every feature, we use as covariates those factors which, in a stepwise method, significantly associate (*P*<0.05/12) with the feature. For the *TasteSensomics* feature, covariates were similarly selected (*P*<0.05/5) among the factors age, sex, BMI, and the first two principal components of the genotype.

We tested the 1,276 features for association in the *CoLaus* cohort with the 713,870 SNPs also measured in the *TasteSensomics* cohort. We pruned the suggestively significant (*P*<5×10^−8^) SNP-feature association pairs by considering two pairs equivalent if their SNPs were in LD (*r^2^*>0.3) and their features were correlated (*r^2^*>0.4). This procedure is an extension of the clumping method implemented in PLINK [49]. We then sought replication in the TasteSensomics cohort [23], [24]. Replication was declared if the discovery and replication effect directions were concordant, the replication P-value was below 0.05/#hits, and the combined association P-value below 5.7×10^−10^. The latter P-value threshold corresponds to the Bonferroni multiple testing correction for both features, where the effective number of tests was estimated [57] to be 125, and SNPs.

To use the admixed genetic background of the *TasteSensomics* cohort for narrowing down the genetic loci giving rise to the association signals, we grouped the replicating SNP-feature associations by genetic loci (1 Mb neighborhood), and ran associations between the implicated feature(s) with all available SNPs in both (discovery and replication) cohorts at the locus. We then meta-analyzed the local association summary statistics (see table S3). The combined results for the strongest association at each locus are reported in table 1.

Features do not directly correspond to the concentration of a single metabolite, so that feature ratios are difficult to interpret. Therefore, in contrast to previous metabolomics association studies, we do not include feature ratios in the first association phase, which substantially reduced the multiple testing burden.

The features involved in replicated associations were subjected to both manual and automated metabolite annotation. Manual annotation was performed using in-house libraries, reference spectra from public databases (HMDB http://www.hmdb.ca, BMRB http://www.bmrb.wisc.edu, Prime http://prime.psc.riken.jp), and the Chenomx NMR Suite software, version 7.1 (Chenomx Inc, Alberta, Canada). Automated annotation was performed by our *metabomatching* method (http://www.unil.ch/cbg), which compares the pseudo-spectrum (see main text) to the spectrum of all metabolites for which a reference spectrum is available in HMDB (to date around 850 metabolites). After pruning correlated spectral bins (to ensure independence) we quantified the similarity between the pseudo-spectrum and the spectrum of a given metabolite by summing up the squared association test statistics
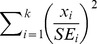
corresponding to the *k* (independent) peaks present in the spectrum of the metabolite. The resulting test-statistic is χ^2^-distributed with *k* degrees of freedom. This allows for obtaining a P-value for having observed as good a match between the pseudo-spectrum and the NMR spectrum as by chance. The procedure is repeated for all metabolites in HMDB, which are then ranked according to their P-values.

For each SNP with confirmed metabolite association, we examined the surrounding 1 Mb window searching for evidence of allelic heterogeneity or imperfect tagging. Within each 1 Mb region, we looked for the best multivariate model (in the sense of AIC) to explain the corresponding metabolic feature in the *CoLaus* sample. If this model provides a significantly better fit to the data than the lead SNP, we attempted to replicate in the *TasteSensomics* cohort. Note that due to the different LD structure in the *CoLaus* and *TasteSensomics* cohorts we did not attempt to replicate the exact same SNPs, but the locus. In case of successful replication we declare the locus to exhibit multiple independent signals. We also attempted fine-mapping of association signals in these regions, using 1000 Genomes imputed genotype association, but no stronger association was revealed.

Mendelian randomization (MR) was carried out by calculating two-stage least squares estimates and comparing them to the direct one stage effect size. We used an *SLC7A9* SNP (rs8101881) as instrument to infer causality between lysine concentration and log transformed age- and sex-corrected eGFR. To verify the assumptions of MR, we noted that the instrument was strongly associated with lysine and, since it is a genotype, is very unlikely to have a common cause with eGFR. The final assumption of MR, namely that all causal effect of the SNP on eGFR is acting through lysine, was examined by verifying that our variables satisfied all of the tests of positive unmeasured confounding (leveraging prior casual assumptions) proposed by Glymour et al. [42]. The selected SNP was not found to be associated with any known confounding factors of eGFR. We used the Durbin-Hausman test [58] to compare the OLS and the 2SLS estimates.

## Supporting Information

Figure S1 Metabolome- and genome-wide association P-values in *CoLaus*. Significant associations (*P_C_*<10^−8^/125) involving features deriving from identified metabolites are shown in color. The carbon-atoms carrying the protons corresponding to the significantly associated features are labeled in the chemical structures.
(PDF)

Figure S2 Additional metabomatching results. Each subfigure shows: (upper half) the NMR spectrum of the control metabolite, and (lower half) the pseudo-spectrum of the *CoLaus* SNPs (linked to the control SNP) with the strongest association to a feature corresponding to one of the peaks of the control metabolite NMR spectrum. (A) N-acetyl-L-lysine: top ranked member of the N-acetylated compound family, vs. rs6546847 in *ALMS1*; (B) Dimethylglycine vs. rs17279437 in *SLC6A20*: while the association of rs17279437 with feature 2.9325 satisfies the threshold for significance in *CoLaus*, the association does not replicate in *TasteSensomics*; (C) Top-ranked compound pair in two-compound metabomatching involving formate, vs. rs4921914 in *NAT2*: rs4921914 is only associated significantly with features which do not correspond to the single peak in the NMR spectrum of formate; (D) 2-hydroxyisobutyrate vs. rs7314056 in *PSMD9*. The metabomatching results for 3-aminoisobutyrate, trimethylamine, lysine, and fucose are shown in the main text.
(PDF)

Figure S3 LD structure in the *FUT2*, *RASIP1* and *IZUMO1* region on chromosome 19. For *CoLaus* (lower triangle), the LD block from rs516246 (ad) to rs11667321 (bh) is associated with fucose, with the strongest association for SNP rs281408 in *RASIP1*. For *TasteSensomics*, the much smaller LD block from rs516246 (ad) to rs633372 (am) is associated with fucose, with the strongest association for SNP rs492602 (ae). The combined association signal mirrors the *TasteSensomics* signal, with again SNP rs492602 showing the strongest association.
(PDF)

Table S1 Details of the 56 SNP-feature associations for which: (1) the discovery P-value, *P_C_*, was below 5×10^−8^, (2) the replication P-value, *P_T_*, was below 0.05/139 (139 associations were found in discovery), (3) the effects matched directions, (4) and the combined P-value obtained by meta-analysis, *P_m_*, was below the Bonferonni threshold of 5×10^−8^/125. Positions are listed according to NCBI build 36; MAF is the minor (effect) allele frequency.
(PDF)

Table S2 Metabomatching testing control SNP-metabolite pairs, and ranking results. Metabomatching tends to perform better in cases involving multi-peak spectra. Control pairs correspond to associations previously discovered in urine metabolome GWAS (from *Nat Genet*, 2011. 43(6): 565–9 and *PLoS Genet*, 2011. 7(9): e1002270), such that: (1) the metabolite is not a ratio; (2) the control association P-value, *P_ref_*, is below 5×10^−8^ (3) the metabolite has a known NMR spectrum; (4) there exists, in *CoLaus*, an association between a (linked) SNP and a feature corresponding to a peak of the control metabolite NMR spectrum with association P-value, *P_C_*, below 10^−6^.
(PDF)

Table S3 Association signal meta-analysis. Association signal meta-analysis. For each locus-metabolite association, the lead SNPs for *CoLaus*, *TasteSensomics*, the cohorts combined, and cohorts from previous studies, are listed, unless the lead SNP is consistent across the four. The published lead SNPs for N-acetylated compounds (rs9309473 in *MolPAGE*), as well as those for trimethylamine (rs7072216 in *MolPAGE*) and 2-hydroxyisobutyrate (rs830124 in *SHIP*) are not part of either the *CoLaus* nor *TasteSensomics* panels but are in perfect LD (*r* ^2^ = *D'* = 1, HapMap Rel 22) with the *CoLaus* lead SNPs rs6546847, rs2147896, and rs7314056, respectively. We therefore consider them equivalent for the purpose of this table. As a result, the trimethylamine and 2-hydroxyisobutyrate associations have a consistent lead SNP and are not listed. Positions are listed according to NBCI build 36. Stars in the *Lead in C; T; m; P* columns indicate whether the SNP is the lead SNP in the respective cohort; a dash in the *Lead in P* column indicates there is no previously published association, in urine. *P___* and *x___* are the P-values and effect sizes for the SNP in *CoLaus*, *TasteSensomics*, and the cohorts combined, respectively. *r_C_^2^* measures the linkage disequilibrium computed with the *CoLaus* genotype between the SNP and the *CoLaus* lead SNP. *r_T_^2^* measures the linkage disequilibrium computed with the *TasteSensomics* genotype between the SNP and the *TasteSensomics* lead SNP. For *AGXT2*, the combined lead SNP is not the shared lead SNP. This can result from the inverse-variance weighting meta-analysis (which assumes common effect sizes) when the associations have different effect sizes across cohorts, as is the case for rs37369. This effect size difference can stem from the differing minor allele frequencies, of 0.08 in *CoLaus* and 0.29 in *TasteSensomics*. The *ALMS1* locus is a good example for how admixed populations can narrow the association signal. While the causal SNP is most probably shared in the two cohorts, a SNP in LD has taken lead of the association signal in *CoLaus* due to stochastic fluctuations or because the causal SNP in unmeasured. This *CoLaus* proxy may not be a proxy in the *TasteSensomics* cohort due to the different LD structures. The meta-analysis attenuates the association signal for this type of SNPs, thereby producing a cleaner signal comprising only SNPs in LD with the causal SNP in *both* cohorts.
(PDF)
