# Supplementary figures and images for: Genome-Wide Association Study of Metabolic Traits Reveals Novel Gene-Metabolite-Disease Links
Source: PLoS Genet. 2014 Feb 20;10(2):e1004132. doi: 10.1371/journal.pgen.1004132 (PMC3930510; doi:10.1371/journal.pgen.1004132)

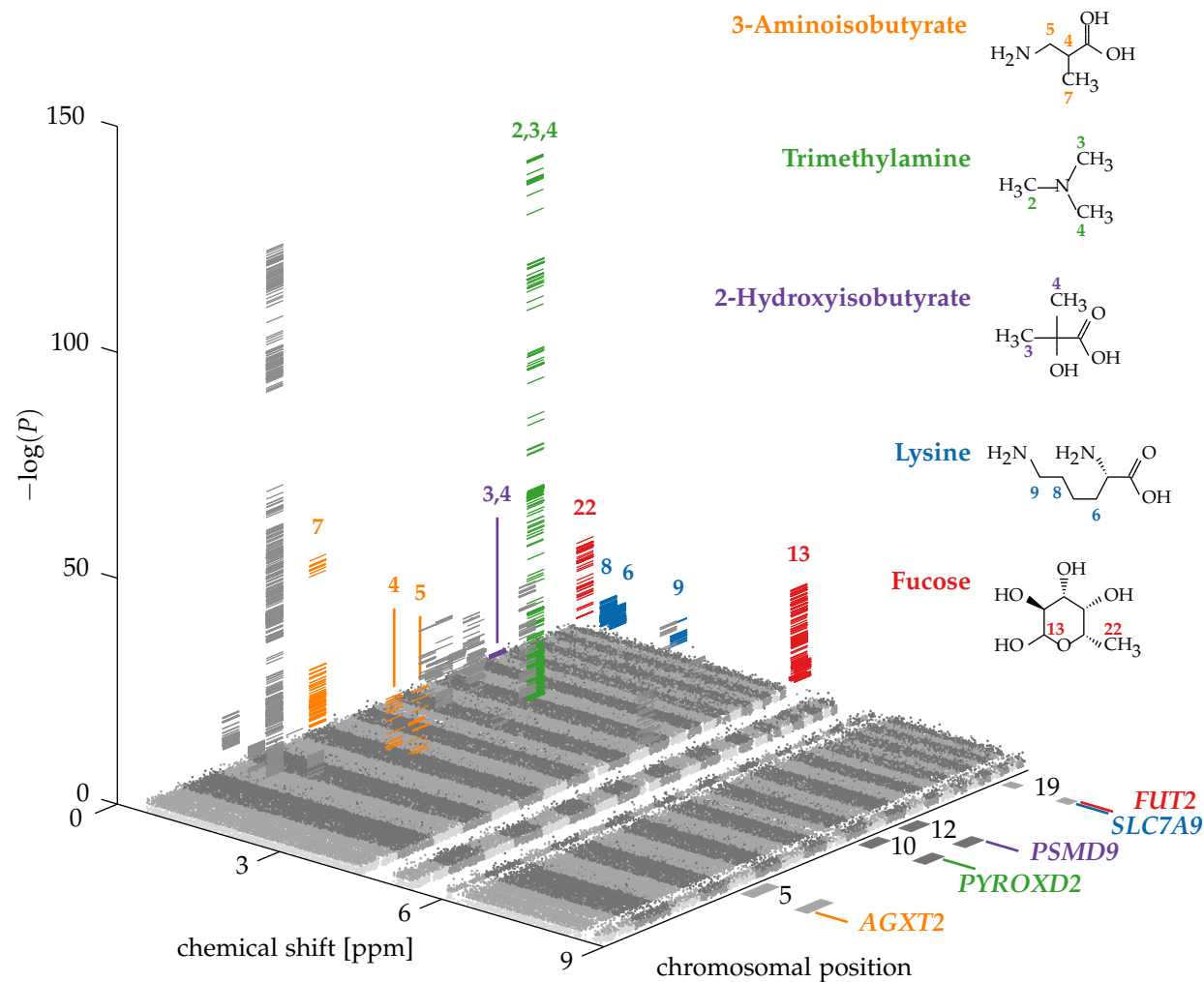

Supplement: Figure S1 — Metabolome- and genome-wide association P-values in CoLaus. Significant associations (PC<10−8/125) involving features deriving from identified metabolites are shown in color. The carbon-atoms carrying the protons corresponding to the significantly associated features are labeled in the chemical structures. (PDF) [file pgen.1004132.s001.pdf]

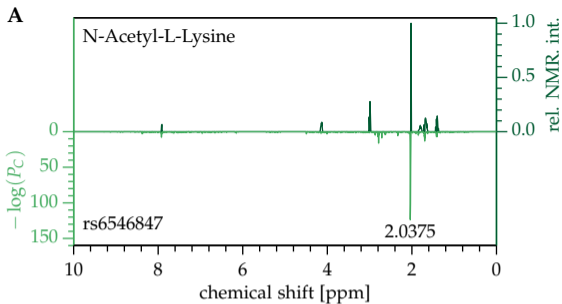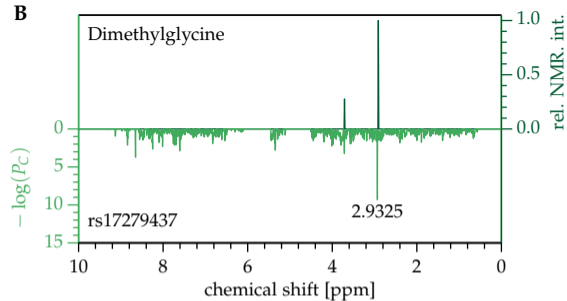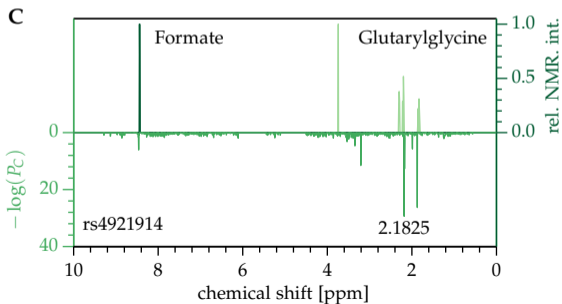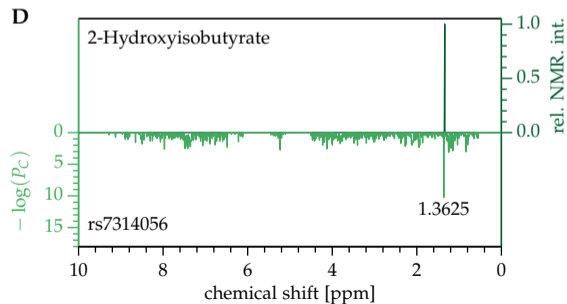

Supplement: Figure S2 — Additional metabomatching results. Each subfigure shows: (upper half) the NMR spectrum of the control metabolite, and (lower half) the pseudo-spectrum of the CoLaus SNPs (linked to the control SNP) with the strongest association to a feature corresponding to one of the peaks of the control metabolite NMR spectrum. (A) N-acetyl-L-lysine: top ranked member of the N-acetylated compound family, vs. rs6546847 in ALMS1; (B) Dimethylglycine vs. rs17279437 in SLC6A20: while the association of rs17279437 with feature 2.9325 satisfies the threshold for significance in CoLaus, the association does not replicate in TasteSensomics; (C) Top-ranked compound pair in two-compound metabomatching involving formate, vs. rs4921914 in NAT2: rs4921914 is only associated significantly with features which do not correspond to the single peak in the NMR spectrum of formate; (D) 2-hydroxyisobutyrate vs. rs7314056 in PSMD9. The metabomatching results for 3-aminoisobutyrate, trimethylamine, lysine, and fucose are shown in the main text. (PDF) [file pgen.1004132.s002.pdf]
