# Supplementary material for: Genome-Wide Association Study of Metabolic Traits Reveals Novel Gene-Metabolite-Disease Links
Source: PLoS Genet. 2014 Feb 20;10(2):e1004132. doi: 10.1371/journal.pgen.1004132 (PMC3930510; doi:10.1371/journal.pgen.1004132)
Supplement: Figure S3 — LD structure in the FUT2, RASIP1 and IZUMO1 region on chromosome 19. For CoLaus (lower triangle), the LD block from rs516246 (ad) to rs11667321 (bh) is associated with fucose, with the strongest association for SNP rs281408 in RASIP1. For TasteSensomics, the much smaller LD block from rs516246 (ad) to rs633372 (am) is associated with fucose, with the strongest association for SNP rs492602 (ae). The combined association signal mirrors the TasteSensomics signal, with again SNP rs492602 showing the strongest association. (PDF) [file pgen.1004132.s003.pdf]

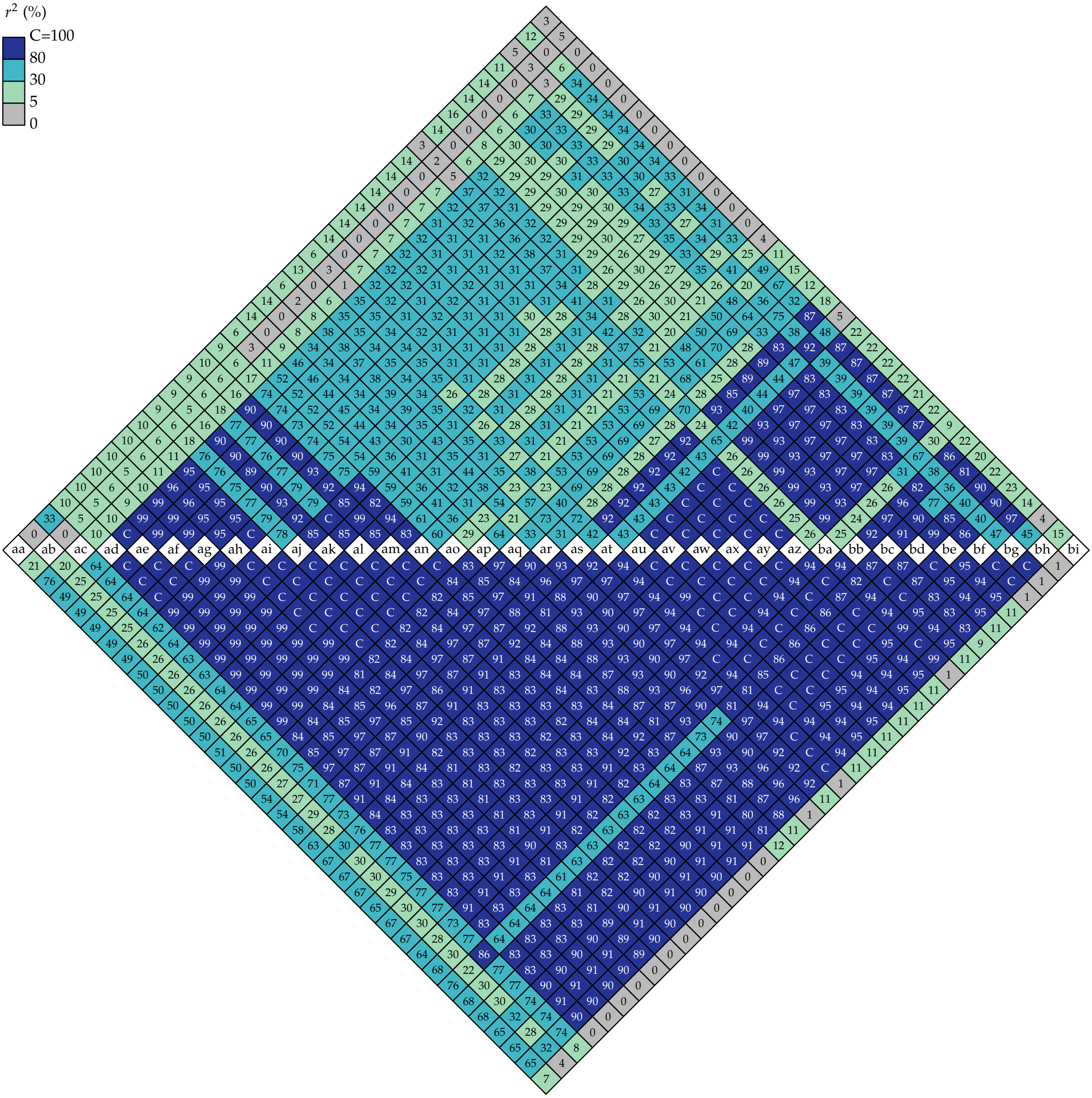

	SNP	Position	Gene
aa	rs3826838	53,875,096	
ab	rs418821	53,891,616	FUT2
ac	rs16982241	53,894,671	FUT2
ad	rs516246	53,897,984	FUT2
ae	rs492602	53,898,229	FUT2
af	rs681343	53,898,274	FUT2
ag	rs601338	53,898,486	FUT2
ah	rs602662	53,898,797	FUT2
ai	rs485186	53,899,018	FUT2
aj	rs504963	53,900,677	FUT2
ak	rs632111	53,900,790	FUT2
al	rs503279	53,900,822	FUT2
am	rs633372	53,901,038	
an	rs676388	53,903,781	
ao	rs281379	53,906,086	
ap	rs281380	53,906,282	
aq	rs281393	53,916,296	RASIP1
ar	rs2287921	53,920,084	RASIP1
as	rs281408	53,925,218	RASIP1
at	rs838147	53,938,678	IZUMO1
au	rs11672046	53,939,413	IZUMO1
av	rs8108136	53,939,505	IZUMO1
aw	rs8108468	53,939,598	IZUMO1
ax	rs12611203	53,940,107	IZUMO1
ay	rs12611211	53,940,143	IZUMO1
az	rs12982115	53,940,452	IZUMO1
ba	rs838145	53,940,542	IZUMO1
bb	rs12975033	53,941,255	IZUMO1
bc	rs12975781	53,941,510	IZUMO1
bd	rs8104897	53,941,626	IZUMO1
be	rs8105137	53,941,700	IZUMO1
bf	rs8106205	53,942,009	
bg	rs838144	53,942,051	
bh	rs11667321	53,942,350	
bi	rs838143	53,943,567	FUT1
