# Supplementary material for: Genome-Wide Association Study of Metabolic Traits Reveals Novel Gene-Metabolite-Disease Links
Source: PLoS Genet. 2014 Feb 20;10(2):e1004132. doi: 10.1371/journal.pgen.1004132 (PMC3930510; doi:10.1371/journal.pgen.1004132)
Supplement: Table S1 — Details of the 56 SNP-feature associations for which: (1) the discovery P-value, PC, was below 5×10^−8^, (2) the replication P-value, PT, was below 0.05/139 (139 associations were found in discovery), (3) the effects matched directions, (4) and the combined P-value obtained by meta-analysis, Pm, was below the Bonferonni threshold of 5×10−8/125. Positions are listed according to NCBI build 36; MAF is the minor (effect) allele frequency. (PDF) [file pgen.1004132.s004.pdf]

				CoLaus				TasteSensomics				Combined	
SNP	Chr	Position	A/B	MAF	Feature	x_C	P_C	MAF	Feature	x_T	P_T	x_m	P_m
ALMS1 - *N*-Acetylated Compounds													
rs12104768	2	73528474	T/C	0.05	2.0375	0.98	8.1×10^{-20}	0.05	2.036	1.09	4.3×10^{-19}	1.03	3.6×10^{-37}
rs11884776	2	73600431	C/T	0.22	1.6975	0.46	2.1×10^{-15}	0.32	1.696	0.23	1.2×10^{-04}	0.35	5.5×10^{-17}
rs6546847	2	73638866	A/G	0.22	2.0375	1.09	1.0×10^{-123}	0.42	2.036	0.71	2.6×10^{-46}	0.91	5.3×10^{-161}
rs6546847	2	73638866	A/G	0.22	2.7875	0.48	7.7×10^{-17}	0.42	2.788	0.21	1.4×10^{-04}	0.34	1.3×10^{-17}
rs4514898	2	73819779	C/T	0.07	2.0375	1.02	2.7×10^{-21}	0.04	2.036	0.58	1.4×10^{-04}	0.87	3.0×10^{-23}
rs3813229	2	73910072	A/G	0.31	2.0375	0.56	2.5×10^{-25}	0.47	2.036	0.31	4.3×10^{-08}	0.44	1.4×10^{-29}
ACADL - Unknown													
rs1509569	2	210840660	A/G	0.44	0.8475	0.40	2.8×10^{-16}	0.41	0.848	0.22	9.6×10^{-05}	0.32	2.7×10^{-18}
AGXT2 - 3-Aminoisobutyrate													
rs16899972	5	35034535	C/A	0.50	1.2025	0.32	1.7×10^{-10}	0.41	1.204	0.22	5.2×10^{-05}	0.27	1.1×10^{-13}
rs7717823	5	35036552	C/T	0.41	1.2025	−0.52	1.2×10^{-22}	0.29	1.204	−0.35	4.0×10^{-09}	−0.44	3.4×10^{-29}
rs7717823	5	35036552	C/T	0.41	3.0975	−0.33	1.8×10^{-09}	0.29	3.096	−0.23	1.1×10^{-04}	−0.29	1.8×10^{-12}
rs468327	5	35038869	G/A	0.21	1.2025	0.66	4.8×10^{-21}	0.27	1.204	0.47	3.3×10^{-15}	0.55	1.1×10^{-33}
rs468327	5	35038869	G/A	0.21	3.0275	0.52	4.1×10^{-13}	0.27	3.028	0.27	8.5×10^{-05}	0.39	4.2×10^{-15}
rs468327	5	35038869	G/A	0.21	3.0975	0.50	4.5×10^{-12}	0.27	3.096	0.34	1.6×10^{-08}	0.41	1.5×10^{-18}
rs468327	5	35038869	G/A	0.21	3.0925	0.43	2.7×10^{-09}	0.27	3.092	0.40	2.0×10^{-11}	0.41	3.4×10^{-19}
rs468327	5	35038869	G/A	0.21	2.6125	0.43	3.5×10^{-09}	0.27	2.612	0.34	4.6×10^{-08}	0.37	1.3×10^{-15}
rs468327	5	35038869	G/A	0.21	2.6075	0.40	3.7×10^{-08}	0.27	2.608	0.34	4.1×10^{-08}	0.36	9.4×10^{-15}
rs37369	5	35072872	C/T	0.08	1.2025	1.26	1.0×10^{-46}	0.29	1.204	0.59	4.6×10^{-28}	0.77	9.7×10^{-64}
rs37369	5	35072872	C/T	0.08	2.6125	0.89	2.6×10^{-21}	0.29	2.612	0.34	7.3×10^{-09}	0.49	3.1×10^{-23}
rs37369	5	35072872	C/T	0.08	3.0975	0.86	3.5×10^{-20}	0.29	3.096	0.45	7.2×10^{-16}	0.56	2.4×10^{-31}
rs37369	5	35072872	C/T	0.08	2.6075	0.85	5.6×10^{-20}	0.29	2.608	0.39	1.1×10^{-11}	0.52	3.4×10^{-26}
rs37369	5	35072872	C/T	0.08	1.1975	0.61	1.9×10^{-10}	0.29	1.196	0.32	3.4×10^{-07}	0.41	7.9×10^{-15}
rs37369	5	35072872	C/T	0.08	3.1075	0.57	3.4×10^{-09}	0.29	3.108	0.36	6.8×10^{-08}	0.43	5.5×10^{-15}
rs37369	5	35072872	C/T	0.08	2.6275	0.53	5.7×10^{-08}	0.29	2.628	0.38	4.4×10^{-10}	0.42	3.0×10^{-16}
rs40200	5	35081502	G/A	0.09	3.0925	0.79	1.4×10^{-24}	0.17	3.092	0.56	7.1×10^{-15}	0.67	9.3×10^{-37}
NAT2 - Unknown													
rs4921914	8	18316718	T/C	0.23	2.1875	0.60	4.3×10^{-21}	0.32	2.188	0.44	2.6×10^{-13}	0.51	4.4×10^{-32}
ABO - Unknown													
rs8176749	9	135121009	C/T	0.07	5.2625	0.56	2.0×10^{-08}	0.11	5.264	0.40	2.1×10^{-05}	0.48	3.7×10^{-12}
rs687289	9	135126927	G/A	0.37	5.1625	−0.30	1.2×10^{-09}	0.37	5.164	−0.22	2.0×10^{-04}	−0.27	1.9×10^{-12}
rs687289	9	135126927	G/A	0.37	4.2375	0.29	3.3×10^{-09}	0.37	4.236	0.25	6.1×10^{-05}	0.27	1.0×10^{-12}
rs657152	9	135129086	C/A	0.39	1.2975	0.36	8.6×10^{-14}	0.38	1.296	0.31	2.2×10^{-07}	0.34	1.3×10^{-19}
rs630014	9	135139543	G/A	0.47	5.1825	−0.37	1.3×10^{-12}	0.48	5.184	−0.30	2.3×10^{-07}	−0.34	2.4×10^{-18}
rs579459	9	135143989	T/C	0.27	2.0525	0.52	9.1×10^{-22}	0.17	2.052	0.44	1.5×10^{-08}	0.49	1.2×10^{-28}
rs579459	9	135143989	T/C	0.27	5.1825	0.52	1.2×10^{-21}	0.17	5.184	0.55	1.9×10^{-12}	0.53	1.8×10^{-32}
rs633862	9	135145265	T/C	0.42	2.0525	−0.34	8.6×10^{-11}	0.49	2.052	−0.23	5.8×10^{-05}	−0.29	5.8×10^{-14}
rs633862	9	135145265	T/C	0.42	5.1825	−0.32	1.1×10^{-09}	0.49	5.184	−0.36	3.3×10^{-10}	−0.34	2.4×10^{-18}
PYROXD2 - Trimethylamine													
rs6584190	10	100114065	T/C	0.10	2.8575	−0.77	9.6×10^{-24}	0.32	2.856	−0.38	2.1×10^{-11}	−0.52	6.4×10^{-30}
rs4345897	10	100137050	A/G	0.34	2.8825	−0.43	1.4×10^{-17}	0.46	2.884	−0.23	7.5×10^{-05}	−0.34	1.7×10^{-19}
rs2147896	10	100138166	A/G	0.35	2.8575	−0.96	1.7×10^{-128}	0.46	2.856	−0.68	5.0×10^{-42}	−0.85	2.6×10^{-164}
rs2147896	10	100138166	A/G	0.35	2.8675	−0.55	2.7×10^{-29}	0.46	2.868	−0.59	4.7×10^{-28}	−0.57	1.6×10^{-55}
rs2147896	10	100138166	A/G	0.35	2.8625	−0.45	1.4×10^{-19}	0.46	2.864	−0.38	5.3×10^{-12}	−0.42	7.9×10^{-30}
rs2147896	10	100138166	A/G	0.35	2.8775	−0.51	7.3×10^{-18}	0.46	2.876	−0.23	5.1×10^{-05}	−0.36	5.9×10^{-19}
rs17455577	10	100155987	C/T	0.31	2.8725	0.56	2.9×10^{-21}	0.22	2.872	0.27	1.4×10^{-04}	0.45	2.7×10^{-22}
PYROXD2 - Unknown													
rs4345897	10	100137050	A/G	0.34	1.7775	−0.30	8.5×10^{-09}	0.46	1.776	−0.24	9.2×10^{-05}	−0.27	4.3×10^{-12}
rs4539242	10	100138048	T/C	0.35	1.8025	−0.41	2.7×10^{-16}	0.46	1.804	−0.28	2.2×10^{-06}	−0.35	1.6×10^{-20}
rs2147896	10	100138166	A/G	0.35	2.7125	−0.58	1.1×10^{-33}	0.46	2.712	−0.26	4.3×10^{-06}	−0.44	4.2×10^{-34}
ACADS - Unknown													
rs3916	12	119661655	G/C	0.28	0.8875	0.46	2.7×10^{-17}	0.22	0.888	0.33	5.0×10^{-07}	0.40	2.4×10^{-22}
PSMD9 - 2-Hydroxyisobutyrate													
rs7314056	12	120827347	C/T	0.13	1.3625	−0.46	6.6×10^{-11}	0.12	1.364	−0.41	1.1×10^{-06}	−0.44	4.0×10^{-16}
SLC7A9 - Lysine													
rs6510300	19	38046832	A/G	0.16	1.7325	0.51	1.5×10^{-15}	0.09	1.732	0.47	1.6×10^{-05}	0.50	1.3×10^{-19}
rs6510300	19	38046832	A/G	0.16	1.9025	0.49	1.4×10^{-14}	0.09	1.904	0.40	2.5×10^{-04}	0.47	2.0×10^{-17}
rs6510300	19	38046832	A/G	0.16	3.0325	0.45	2.8×10^{-12}	0.09	3.032	0.44	7.9×10^{-05}	0.45	1.0×10^{-15}
rs8101881	19	38056468	T/C	0.42	3.0325	0.39	5.3×10^{-15}	0.45	3.032	0.54	3.8×10^{-21}	0.45	1.2×10^{-33}
rs8101881	19	38056468	T/C	0.42	1.7325	0.36	7.4×10^{-13}	0.45	1.732	0.56	4.6×10^{-24}	0.45	1.3×10^{-33}
rs8101881	19	38056468	T/C	0.42	1.9025	0.35	2.4×10^{-12}	0.45	1.904	0.56	1.6×10^{-24}	0.44	2.0×10^{-33}

table continues on next page

				CoLaus				TasteSensomics				Combined	
SNP	Chr	Position	A/B	MAF	Feature	x_C	P_C	MAF	Feature	x_T	P_T	x_m	P_m
FUT2 - Fucose													
rs2287921	19	53920084	C/T	0.48	5.2125	0.58	2.2×10^{-30}	0.34	5.212	0.34	1.4×10^{-09}	0.48	3.1×10^{-36}
rs2287921	19	53920084	C/T	0.48	5.2825	0.41	2.0×10^{-14}	0.34	5.284	0.30	2.5×10^{-06}	0.37	6.7×10^{-19}
rs2287921	19	53920084	C/T	0.48	5.2275	0.33	2.2×10^{-09}	0.34	5.228	0.25	9.6×10^{-05}	0.30	1.4×10^{-12}
rs281408	19	53925218	C/A	0.45	1.2575	0.50	9.5×10^{-27}	0.43	1.256	0.31	2.3×10^{-08}	0.42	3.9×10^{-32}
