# Supplementary material for: Genome-Wide Association Study of Metabolic Traits Reveals Novel Gene-Metabolite-Disease Links
Source: PLoS Genet. 2014 Feb 20;10(2):e1004132. doi: 10.1371/journal.pgen.1004132 (PMC3930510; doi:10.1371/journal.pgen.1004132)
Supplement: Table S2 — Metabomatching testing control SNP-metabolite pairs, and ranking results. Metabomatching tends to perform better in cases involving multi-peak spectra. Control pairs correspond to associations previously discovered in urine metabolome GWAS (from Nat Genet, 2011. 43(6): 565–9 and PLoS Genet, 2011. 7(9): e1002270), such that: (1) the metabolite is not a ratio; (2) the control association P-value, Pref, is below 5×10^−8^ (3) the metabolite has a known NMR spectrum; (4) there exists, in CoLaus, an association between a (linked) SNP and a feature corresponding to a peak of the control metabolite NMR spectrum with association P-value, PC, below 10−6. (PDF) [file pgen.1004132.s005.pdf]

SNP (control)	Gene	Metabolite	P_{ref}	Spectrum	Cohort	P_C	Rank
rs37369	*AGXT2*	3-aminoisobutyrate	3.2×10^{-75}	multi-peak	SHIP	1.0×10^{-46}	1
rs8101881	*SLC7A9*	lysine	1.0×10^{-8}	multi-peak	SHIP	1.5×10^{-15}	1
rs17279437	*SLC6A20*	dimethylglycine	5.5×10^{-10}	multi-peak	SHIP	4.6×10^{-10}	3
rs11884776	*ALMS1*	N-acetylated compounds	4.1×10^{-19}	multi-peak	MolPAGE	1.0×10^{-123}	3, 14, 21
rs4539242	*PYROXD2*	trimethylamine	2.8×10^{-23}	single peak	MolPAGE	1.7×10^{-128}	44
rs830124	*PSMD9*	2-hydroxyisobutyrate	1.6×10^{-15}	single peak	SHIP	6.6×10^{-11}	65
rs1495743	*NAT2*	formate	1.4×10^{-10}	single peak	SHIP	6.0×10^{-7}	222 (9)
