# Supplementary material for: Genome-Wide Association Study of Metabolic Traits Reveals Novel Gene-Metabolite-Disease Links
Source: PLoS Genet. 2014 Feb 20;10(2):e1004132. doi: 10.1371/journal.pgen.1004132 (PMC3930510; doi:10.1371/journal.pgen.1004132)
Supplement: Table S3 — Association signal meta-analysis. Association signal meta-analysis. For each locus-metabolite association, the lead SNPs for CoLaus, TasteSensomics, the cohorts combined, and cohorts from previous studies, are listed, unless the lead SNP is consistent across the four. The published lead SNPs for N-acetylated compounds (rs9309473 in MolPAGE), as well as those for trimethylamine (rs7072216 in MolPAGE) and 2-hydroxyisobutyrate (rs830124 in SHIP) are not part of either the CoLaus nor TasteSensomics panels but are in perfect LD (r 2 = D' = 1, HapMap Rel 22) with the CoLaus lead SNPs rs6546847, rs2147896, and rs7314056, respectively. We therefore consider them equivalent for the purpose of this table. As a result, the trimethylamine and 2-hydroxyisobutyrate associations have a consistent lead SNP and are not listed. Positions are listed according to NBCI build 36. Stars in the Lead in C; T; m; P columns indicate whether the SNP is the lead SNP in the respective cohort; a dash in the Lead in P column indicates there is no previously published association, in urine. P_ and x_ are the P-values and effect sizes for the SNP in CoLaus, TasteSensomics, and the cohorts combined, respectively. rC2 measures the linkage disequilibrium computed with the CoLaus genotype between the SNP and the CoLaus lead SNP. rT2 measures the linkage disequilibrium computed with the TasteSensomics genotype between the SNP and the TasteSensomics lead SNP. For AGXT2, the combined lead SNP is not the shared lead SNP. This can result from the inverse-variance weighting meta-analysis (which assumes common effect sizes) when the associations have different effect sizes across cohorts, as is the case for rs37369. This effect size difference can stem from the differing minor allele frequencies, of 0.08 in CoLaus and 0.29 in TasteSensomics. The ALMS1 locus is a good example for how admixed populations can narrow the association signal. While the causal SNP is most probably shared in the two cohorts, a SNP in [file pgen.1004132.s006.pdf]

SNP	Chr	Position	Lead In C	T	m	P_C	x_C	P_T	x_T	P_m	x_m	r_C^2 vs. lead	r_T^2 vs. lead
ALMS1 - N-Acetylated Compounds (2.0375)													
rs6546847	2	73638866	⋆			1.0×10^{-123}	1.09	2.6×10^{-46}	0.71	5.3×10^{-161}	0.91	1.00	0.01
rs11884776	2	73600431		⋆	⋆	1.1×10^{-117}	1.08	3.6×10^{-94}	0.96	3.4×10^{-209}	1.02	0.94	1.00
ACADL - Unknown (0.8475)													
rs1509569	2	210840660	⋆			2.8×10^{-16}	0.40	9.6×10^{-05}	0.22	2.7×10^{-18}	0.32	1.00	0.67
rs3764913	2	210783154		⋆	⋆	3.7×10^{-16}	0.41	3.3×10^{-05}	0.27	2.9×10^{-19}	0.36	0.67	1.00
AGXT2 - 3-Aminoisobutyrate (1.2025)													
rs37369	5	35072872	⋆	⋆		1.0×10^{-46}	1.26	4.6×10^{-28}	0.59	9.7×10^{-64}	0.77	1.00	1.00
rs37370	5	35075243			⋆	9.7×10^{-46}	1.05	1.2×10^{-22}	0.81	1.2×10^{-65}	0.94	0.87	0.13
NAT2 - Unknown (2.1875)													
rs4921914	8	18316718	⋆		⋆	4.3×10^{-21}	0.60	2.6×10^{-13}	0.44	4.4×10^{-32}	0.51	1.00	0.72
rs4646250	8	18304878		⋆		6.7×10^{-14}	0.46	1.3×10^{-14}	0.44	6.0×10^{-27}	0.45	0.77	1.00
PYROXD2 - Unknown (1.8025)													
rs4539242	10	100138048	⋆			2.7×10^{-16}	-0.41	2.2×10^{-06}	-0.28	1.6×10^{-20}	-0.35	1.00	0.83
rs4345897	10	100137050		⋆	⋆	4.0×10^{-16}	-0.41	6.7×10^{-07}	-0.29	4.5×10^{-21}	-0.36	0.98	1.00
ACADS - Unknown (0.8875)													
rs3916	12	119661655	⋆		⋆	2.7×10^{-17}	0.46	5.0×10^{-07}	0.33	2.4×10^{-22}	0.40	1.00	0.45
rs2239760	12	119647901		⋆		1.6×10^{-11}	0.34	2.6×10^{-07}	0.27	3.4×10^{-17}	0.31	0.46	1.00
FUT2 - Fucose (1.2575)													
rs281408	19	53925218	⋆			9.5×10^{-27}	0.50	2.3×10^{-08}	0.31	3.9×10^{-32}	0.42	1.00	0.57
rs492602	19	53898229		⋆	⋆	8.6×10^{-23}	0.71	1.3×10^{-23}	0.54	6.9×10^{-44}	0.60	0.87	1.00
